# Semi-supervised assisted multi-task learning for oral optical coherence tomography image segmentation and denoising

**DOI:** 10.1364/BOE.545377

**Published:** 2025-02-26

**Authors:** Jinpeng Liao, Tianyu Zhang, Simon Shepherd, Michaelina Macluskey, Chunhui Li, Zhihong Huang

**Affiliations:** 1 School of Science and Engineering, University of Dundee, DD1 4HN, Scotland, UK; 2 Healthcare Engineering, School of Physics and Engineering Technology, University of York, UK; 3 School of Dentistry, University of Dundee, Dundee, DD1 4HN, Scotland, UK

## Abstract

Optical coherence tomography (OCT) is promising to become an essential imaging tool for non-invasive oral mucosal tissue assessment, but it faces challenges like speckle noise and motion artifacts. In addition, it is difficult to distinguish different layers of oral mucosal tissues from gray level OCT images due to the similarity of optical properties between different layers. We introduce the Efficient Segmentation-Denoising Model (ESDM), a multi-task deep learning framework designed to enhance OCT imaging by reducing scan time from ∼8s to ∼2s and improving oral epithelium layer segmentation. ESDM integrates the local feature extraction capabilities of the convolution layer and the long-term information processing advantages of the transformer, achieving better denoising and segmentation performance compared to existing models. Our evaluation shows that ESDM outperforms state-of-the-art models with a PSNR of 26.272, SSIM of 0.737, mDice of 0.972, and mIoU of 0.948. Ablation studies confirm the effectiveness of our design, such as the feature fusion methods, which enhance performance with minimal model complexity increase. ESDM also presents high accuracy in quantifying oral epithelium thickness, achieving mean absolute errors as low as 5 µm compared to manual measurements. This research shows that ESDM can notably improve OCT imaging and reduce the cost of accurate oral epithermal segmentation, improving diagnostic capabilities in clinical settings.

## 1. Introduction

Oral mucosa protects the oral cavity, serves as the barrier against external harmful substances, while the labial and buccal regions are common sites for malignancies [1]. Clinically, breaches in the oral mucosa appear as erosions or ulcers, often presenting as greyish-white patches due to fibrin deposition [2]. While many oral ulcers are benign and self-resolving, some indicate serious conditions like infections, autoimmune diseases, trauma, or malignancies [3]. Early oral cancer diagnosis can improve survival rates by enabling prompt, precise treatment, showing the urgent need for effective tools in oral mucosal assessment.

Biopsy is the gold standard in oral mucosal diagnosis but is inherently invasive, involving incision to obtain tissue causing patient discomfort. Non-invasive imaging methods like intra-oral scanner [4] and mucoscopy [5], offer early detection but have limited penetration depth. Thus, there is an urgent need for non-invasive imaging techniques that can provide accurate assessments of oral tissues with high-resolution and providing depth-information. Optical coherence tomography (OCT) is a non-invasive imaging modality that provides micro-anatomical visualization with an axial resolution of ∼10 µm and a penetration depth of 1-2 mm [6]. Recent studies indicate that OCT can differentiate between normal and abnormal tissues, showing its potential as a non-invasive alternative to biopsy in oral mucosal assessments [7].

However, speckle noise, an inherent byproduct of optical coherence, significantly degrades oral OCT image quality by reducing contrast and blurring tissue structures, compromising diagnostic integrity [8]. Hence, effectively addressing speckle noise is necessary to maintain the accuracy of OCT assessments in oral disease. While a series of algorithms have been explored to enhance the OCT image quality, each has its inherent trade-offs. The frame-averaging method is based on a high-repetition OCT scan (e.g., 4-6 repetitions) to reduce the noise in OCT image [9]. However, this method would increase the OCT scan duration, inherently increasing the risk of motion artifacts in OCT imaging, especially with uncooperative patients or when scanning hard-to-reach areas, such as the buccal mucosa area. The other methods, such as wavelet-based transform [10] and noise adaptive wavelet thresholding [11], are risking insufficient or excessive smoothing. Adaptive speckle suppression filters offer a tailored approach but may not be universally effective across all OCT image types, occasionally blurring essential features [12]. These challenges show the need for a robust algorithm that can effectively reduce speckle noise without degrading the diagnostic value of oral OCT images.

Segmentation is essential in oral OCT imaging, as it enables the differentiation of various oral layers and facilitates detailed analysis, aiding in the detection and localization of pathological changes. Accurate segmentation of the oral epithelium using OCT allows for the detection of subtle thickness and structural changes, which are essential for identifying and differentiating between benign and malignant oral lesions [13,14]. However, distinguishing between oral epithelium and stroma is challenging due to the similar optical properties of these layers. Hence, accurately annotating the oral tissues based on OCT remains a difficult task, even for experts [15]. Besides, manual annotation is time-consuming and subject to variability in interpretation, repeatability, and interobserver agreement, making it difficult for clinical applications. Conventional OCT layer segmentation methods, such as Shapelet-based [16] and intensity-based [17] methods, are heavily rely on image quality, struggling with artifacts and speckle noise [18]. Additionally, the epithelial layer's thickening causes the transitionary zone to approach the OCT system's maximum imaging depth, where declining signal-to-noise ratios and increased light attenuation complicate layer delineation [19]. These limitations underscore the need for advanced segmentation methods to manage image variability and noise, ensuring accuracy and reliability.

Thus, OCT-based segmentation and denoising are essential tasks for OCT to use in oral mucosa applications. Recently, convolutional neural networks (CNNs) have shown impressive results in achieving these tasks. In OCT image denoising, Bin et al. [20] introduced a denoising CNN (DnCNN) model that effectively reduces noise and enhances image clarity for retinal OCT images. Based on DnCNN, Maryam et al. [21] developed a deep feature loss function to further refine retinal OCT image denoising performance, while Zhao et al. [22] proposed the SM-GAN, which incorporates 16 residual blocks and surpasses the SRGAN [23] in denoising efficacy. In skin OCT images, U-Net-based methods [24] have improved the efficiency of the skin layer segmentation [25]. In addition, Mao et al. [26] proposed a U-Net-based method for denoising retinal OCT images, improving the segmentation accuracy of the anterior lamina cribrosa. Kepp et al. [27] further proposed a densely connected (DC)U-Net for enhanced feature reuse, showing better performance in mouse skin layer segmentation. Our previous work proposed an LS-Net to segment the skin epidermis/dermis layer with a high accuracy while reducing the model complexity [28]. Nevertheless, these approaches share common limitations. First, existing methods primarily target mouse skin or skin OCT images, differing significantly from oral OCT in tissue characteristics. Second, these segmentation methods require large-scale labeled datasets, with accuracy and generalizability constrained by limited data [29]. Third, they often focus solely on segmentation or denoising tasks, requiring separate training for each task. Thus, there is a need for efficient multi-task models in oral OCT image processing. Fourth, CNNs are limited by small receptive fields and localized convolutional operations, resulting in hard-to-capture long-term information, which is crucial for accurately modeling oral tissue morphology.

To address the current challenges in intraoral OCT imaging, we propose the oral OCT-based Efficient Segmentation-Denoising Model (ESDM). By integrating denoising and segmentation tasks into a single, efficient framework, ESDM simultaneously reduces speckle noise and segments the oral epithelium and stroma layers based on a fast one-repetition OCT scan. This integration allows us to obtain high-quality, low-noise intraoral OCT images and accurate segmentation label for oral epithelium thickness calculation, while significantly reducing the scan time from approximately 8 seconds to 2 seconds with our lab-built 200 kHz swept-source OCT system. The shortened scan time minimizes motion artifacts, which is particularly important when scanning hard-to-reach areas like the buccal mucosa.

ESDM utilizes a shared encoder with separate decoders for segmentation and denoising, avoiding redundant learning of common features and improving generalization while reducing memory consumption. Inspired by Wu et al.[30], we introduced convolutional operations into the transformer architecture, providing spatial relationship information for the multi-head self-attention mechanism. This enhancement improves ESDM's performance in both segmentation and denoising tasks. Besides, ESDM improves accurate segmentation for epithelium thickness quantification, thereby improving the clinical value of OCT imaging devices in oral healthcare.

Our contributions are as follows: (1) novel multi-task architecture: We present ESDM, the deep learning-based method for simultaneous segmentation and denoising of one-repetition oral OCT images, addressing the challenges of speckle noise and motion artifacts in intraoral imaging. (2) semi-supervised learning approach: To enhance segmentation effectiveness with limited manual labels, we propose a semi-supervised assisted multi-task deep learning pipeline that utilizes pseudo-labels, reducing the need for extensive manual annotation. (3) reduce scan time: By enabling high-quality imaging with a one-repetition scan, ESDM reduces the sampling time by 75%, from approximately 8 seconds to 2 seconds, decreasing the risk of motion artifacts and enhancing patient comfort. (4) comprehensive evaluation: We did experiments and ablation studies to evaluate ESDM, demonstrating faster inference times and better segmentation and denoising performance compared to existing models.

## 2. Related works

### 2.1. Vision transformer in image segmentation and denoising

By utilizing self-attention and flattening the image as a sequence of patches, vision transformer (ViT) [31] offers the advantage of capturing long-term dependencies within an image, which allows for more flexible and often more accurate representations compared to the local and fixed receptive fields of CNNs [32]. With the hierarchical shifted windows (Swin), Swin-transformers exceed the ViT in image classification [33]. Besides, Wu et al. [30] introduced the convolutional projection to the transformer (CvT), integrating spatial information when converting image patches into sequences, thereby enhancing image feature extraction capabilities compared to ViT.

In image denoising, Liang et al. [34] proposed SwinIR, which reconstructs high-quality images from noisy and blurry inputs. By using a novel locally enhanced window transformer and extracting features at multiple scales, Wang et al. proposed UFormer [35], which is more efficient than SwinIR, achieving better performance with less model complexity.

In medical image segmentation (MIS), TransUNet [36] combines the advantages of U-Net and ViT, leveraging a global receptive field to enhance long-term information processing beyond CNNs. Swin-UNet [37] improves efficiency and scalability with shifted window mechanisms, outperforming TransUNet. Additionally, SegFormer [38] utilizes an efficient self-attention mechanism with convolution layers for query and key sequence reduction, reducing model complexity while maintaining high performance in MIS.

However, ViT utilizes linear projection layers for sequence generation for the multi-head attention mechanism, which does not account for the spatial relationships between image patches, resulting in ViT struggling to capture local features as effectively as CNNs. Additionally, training and inference of ViT models demand high computational resources and memory, impacting their efficiency and practicality. Moreover, ViT models require large datasets, such as JFT-300 M, posing challenges for oral OCT image denoising and segmentation due to the difficulty of acquiring high-quality scans with accurate segmentation labels.

### 2.2. Multi-task deep learning

Multi-task deep learning (MTDL) is an algorithm that enhances model performance by integrating information from various tasks through their data, learning a shared feature representation [39]. MTDL allows simultaneous training by sharing a common feature representation across different tasks. MTDL primarily uses two methods: hard-parameter sharing and soft-parameter sharing [40]. Soft parameter sharing uses separate models for each task with regularization terms to encourage parameter similarity, offering greater flexibility for tasks with different requirements but increasing computational resources and model complexity. Hard-parameter sharing, the most common MTDL approach in neural networks, shares hidden layers among tasks while maintaining separate task-specific output layers, and reducing the risk of overfitting [41].

Although MTDL has been widely used in medical image processing and analysis, their application to oral OCT image segmentation and denoising remains underexplored [42]. Most existing MTDL models use the same decoder for both tasks. For instance, Ye et al. introduced a co-learning deep learning framework for visible light-OCT image denoising and segmentation [43], demonstrating enhanced segmentation accuracy and robustness with limited annotated data. In addition, Buchholz et al. proposed a joint denoising and segmentation method that uses self-supervised learning to improve segmentation performance with limited annotated data [44]. However, recent studies indicate that segmentation decoders with fewer parameters and reduced complexity can achieve good performance while improving efficiency [38,45]. Further research is needed for MTDL training and to develop more efficient methods to minimize computational resource waste for simultaneous segmentation and denoising of oral OCT images.

## 3. System and data pre-processing

### 3.1. Swept-source OCT system

**Fig. 1.**
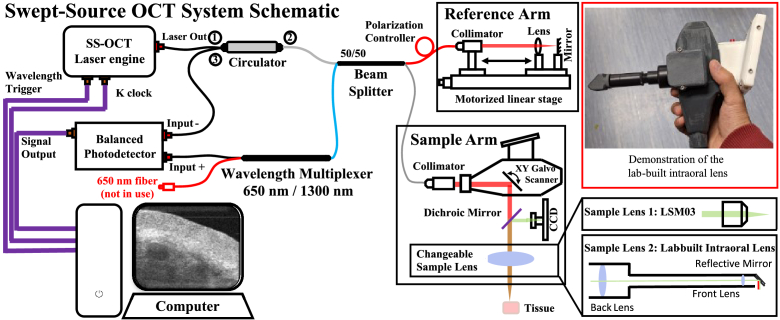
The schematic of the SSOCT system used in this study. The data acquisition card used on the computer is an ATS-9371 from AlazarTech Inc. The sample arm has a changeable sample lens to examine different sites of the oral cavity. The sample arm is hand-held with a flexible design. Sample lens 1 is an LSM03 from Thorlabs Inc. with a 35 mm focal length. Sample lens 2 is a lab-built intraoral lens for difficult-to-reach site data acquisition. The red box demonstrates the flexible handheld scan probe with intraoral lens [46].

A lab-built swept-source OCT (SSOCT) system was utilized to non-invasively acquire the oral structure with a hand-held and flexible probe, as shown in Fig. 1.

The swept-source laser (SL132120 from Thorlabs Inc.) in this system has a wavelength of 1310 nm, a bandwidth of 100 nm, and a 200 kHz A-scan rate. This system has a theoretical axial resolution of ∼7.4 µm in air, with a penetration depth of ∼2 mm. The axial conversion coefficient of this SSOCT system is ∼8.74 µm/pixel in air in the cross-sectional images. The system lateral resolution with LSM03 is ∼ 19.68 µm in air, and ∼ 39.38 µm with intraoral lens. More details about the system with the intraoral sample lens can be found in [46], and the more details of the system with the LSM03 sample lens are in [47].

### 3.2. Data collection

To develop a comprehensive multi-task model for oral OCT image denoising and segmentation, dataset was collected using two different lenses, as shown in Fig. 1. The scan positions are the oral lip and intraoral side. With the LSM03 lens, 64 volumes of OCT data were collected from 19 participants, including 60 lip data and 4 intraoral side data. With the intraoral lens, 86 volumes of OCT data were collected from an additional 27 participants, including 66 lip data and 20 intraoral side data. More details on data collection with these two lenses are available in our previous works [46,48]. The data collection was approved by the School of Science and Engineering Research Ethics Committee of the University of Dundee, in accordance with the tenets of the Declaration of Helsinki.

Regarding the scanning protocol, using the LSM03 sample lens, each OCT scan comprised one OCT data cube with a pixel size of 600 × 600 × 960 × 4 (X-transverse axis, Y-transverse axis, Z-axial axis, and NR-repetitions of OCT scan), and each OCT scan took about 8 seconds. The scanning range is 5.16 × 5.16 mm^2^. When using the intraoral sample lens, each OCT scan comprised one OCT data cube with a pixel size of 400 × 400 × 960 × 3 (X, Y, Z, N), and each scan took about 4 seconds. The scanning range is 5.2 × 5.2 mm^2^.

To ensure stability during handheld OCT data collection, operators used support points and repeated each scan three times, selecting the data with the least motion. Besides, the focusing of the data collection is achieved by adjusting the distance between the sample lens and the sample tissue manually before the data acquisition.

### 3.3. Data pre-processing

In the image denoising task, the frame-averaging (FA) [9] was utilized to generate high-quality OCT structure images (ground-truth) based on the N-repetition of acquired OCT signals. The frame-averaging algorithm can reduce the speckle noise that originates from the coherent nature of the light source. The formulation of the frame-averaging algorithm can be written as (1):

(1) $$V_{frame-averaged}=\frac{1}{NR}\sum_{f=1}^{F}\left(V_{1}^{f}+V_{2}^{f}+\ldots +V_{NR}^{f}\right)$$

where NR is the number of repetitions in the OCT data, F is the total number of B-frame in the OCT data, and f is the current frame for frame-averaging calculation. To ensure alignment of the low-quality OCT images with the high-quality ground-truth OCT images, the first repetition of OCT data was used to generate the OCT images as model input. After obtaining the counterpart noisy input and high-quality OCT data, the OCT data were split into 2D B-frame images with a shape of 600 × 960 (LSM03 lens) and 400 × 960 (intraoral lens). To extract image patches from the OCT data, we employed a crop box measuring 256 × 256 pixels along the X-transverse and Z-axial axes. Given the OCT device's penetration depth of approximately 2 mm, an axial dimension of 256 pixels was sufficient to capture most structural signals. The transverse axis cropping ranges were selected based on the lens used during data collection. Specifically, for B-frames obtained with the intraoral lens, we used cropping ranges of [0,256] and [144,400]. For B-frames collected with the LSM03 lens, the cropping ranges were [0,256], [256,512], and [344,600]. Additionally, the shape of 256 × 256 helps prevent out-of-memory issues during the model training due to limited memory and computational costs.

**Fig. 2.**
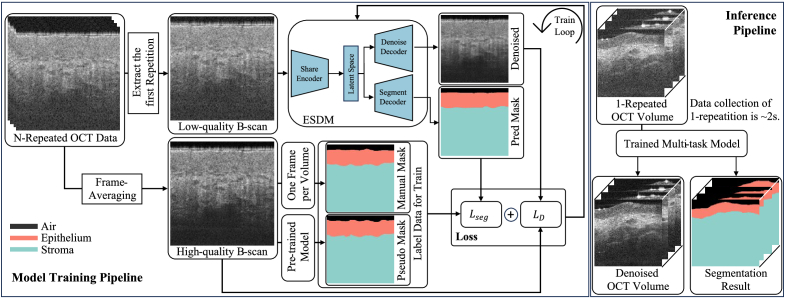
The Pipeline of Multi-task Deep Learning Model Training and Inference.

For semi-supervised training, an LS-Net model pre-trained on a skin OCT dataset was used to generate pseudo labels based on the high-quality images from the frame-averaging method, as shown in Fig. 2. Details of the pre-trained LS-Net model can be found in our previous study [29]. To provide high-precision labels for model training and reduce the cost of manual labeling, the first frame of the OCT volume was manually labeled. For model evaluation, B-frames and paired labels were manually labeled by two experts using custom software in Python. These B-frames were sampled from independent OCT volumes, distinct from the training data, at a rate of 40 frames per volume.

In total, 126 volumes were used to create the training and validation set for model training, and the remaining 24 volumes (6 oral sides and 18 lips) were used to generate the test set for model performance evaluation. Using the bundle of input noisy OCT images, high-quality OCT images, and pseudo labels as a data pair, 90780 pairs were used for the training set, and 22693 pairs were used for the validation set to prevent overfitting. For the test set, 18636 pairs of low- and high-quality OCT images were used for denoising performance evaluation, and 1125 pairs of OCT images and manual labels were used for image segmentation evaluation and epithelium thickness evaluation.

## 4. Multi-task deep learning model

### 4.1. Model training and inference pipeline

A pipeline of the ESDM training and inference is shown in Fig. 2, including the loss calculation for segmentation and denoising tasks, respectively. In the model inference stage, the OCT data collection procedure is facilitated from ∼8s to ∼2s. The one-repeated OCT signal is directly processed by the trained ESDM, then outputs the denoised OCT image and the mask containing the segmentation information of the oral epithelium and stroma.

### 4.2. Network architecture

**Fig. 3.**
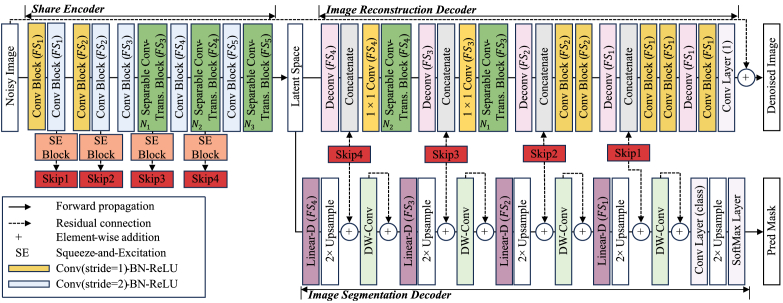
The proposed Efficient Segmentation-Denoising Model (ESDM) architecture. The model is mainly consist of convolution block, separable convolutional transformer block, linear decoder (Linear-D), squeeze-and-excitation (SE) block, deconvolution (Deconv) layer, and depth-wise convolution (DW-Conv) layer. $FS_{1}$ to $FS_{4}$ are the filter size setting in various depths of model. $N_{1}$ to $N_{3}$ are the number of separable convolutional transformer block in each stage.

The architecture of the proposed ESDM is in Fig. 3. ESDM has a share encoder with separate decoders for image denoising and segmentation, respectively. Similar to U-Net model [24], the shared encoder and decoders have the five downsample and up-sample stages to better extract the high-level features from the input noisy image. While CvT has been shown to improve model denoising performance, it increases model complexity and computational demand compared to the pure-ViT [49].

To reduce model complexity and computational demand, two convolution blocks with a stride of 2 are utilized to reduce the size of the feature map. These blocks offer two advantages: 1) they provide spatial information among image patches for the subsequent separable convolutional transformer block. 2) they reduce the computational demand of the model. The squeeze-and-excitation (SE)-block is utilized to effectively integrate the shallow-to-deep features in the following image denoising and segmentation decoders [50]. Inspired by Su et al. [45], ESDM utilizes a lightweight segmentation decoder instead of the same architecture as the denoising decoder, reducing the complexity.

#### 4.2.1. Separable convolutional transformer layer

Compared to the vanilla vision transformer layer [31] and the convolutional transformer (CvT) [30], the separable convolutional transformer (SCT) layer utilizes a 1 × 1 convolution layer with a 3 × 3 depth-wise convolution layer to generate the query (Q), key (K), and value (V) sequences for the multi-head self-attention mechanism. Compared to the CvT-based model [51] that utilized convolution layers for Q, K, V sequences generation, the SCT layer maintains the global receptive field of transformer while extracting both spatial and channel-wise features.

**Fig. 4.**
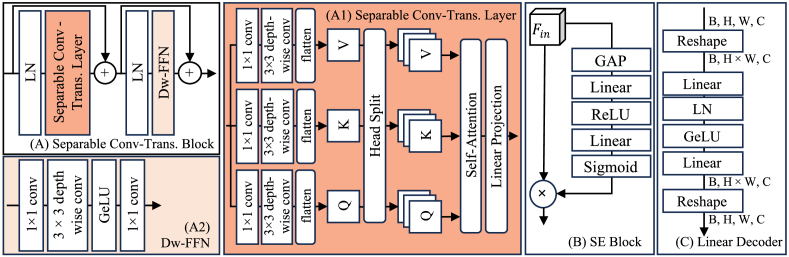
The schematic of the component in the ESDM model. (A) Separable convolutional transformer block. (A1) separable convolutional transformer layer. (A2) depth-wise feed forward network (DW-FFN). (B) Squeeze-and-Excitation (SE) block. (C) linear decoder block. LN: layer normalization; GAP: global averaging pooling; ×: element-wise production.

As shown in Fig. 4(A1), the SCT layer can be seen as the combination of three stages, including Q, K, V sequences extraction, sequences head split, and self-attention mechanism. Taking the input of SCT layer with a shape of H × W × C, the forward processing of the Q, K, V sequences extraction can be written as:

(2) $$\text{Sequenc}{\text{e}}_{\text{Q},\text{K},\text{V}}=\text{flatten}(\text{DW}(\text{Con}{\text{v}}_{1\times 1}({\text{x}}_{\text{input}})))$$

where DW is depth-wise convolution layer with a kernel size of 3 × 3, $\text{Con}{\text{v}}_{1\times 1}$ has a same filter size with the input, i.e., C. Flatten is a reshape operation that can reshape the output from H × W × C to HW × C. After obtaining the Q, K, V sequences, the head split operation is used to reshape the sequences from HW × C to HW/head × head × C for the following multi-head self-attention operation. The self-attention (SA) mechanism can be written as:

(3) $$\text{SA}(\text{Q},\text{K},\text{V})=\text{Softmax}(\text{Q}{\text{K}}^{\text{T}}/\text{d})\text{V}$$

where T is the transposing operation that applied on sequence K. d is a dimensionality of the Q sequence, which is used as scaling factor to prevent large dot-product value from QK, thereby producing stable gradient and improving training procedure. After processing by the SA, the output is a sequence with a shape of HW/head × head × C. That sequence is then reshaped to HW × C, and then processed by linear projection layer with a unit of C, and the output of SCT layer is a 2D sequence with a shape of HW × C.

#### 4.2.2. Depth-wise feed forward network

Zamir et al. [49] experiment indicated that the integration of convolution layers into the feed-forward network (FFN) enhances image reconstruction performance with transformer while maintaining model efficiency. The depth-wise (DW)-FFN employs pointwise (PW) and depth-wise (DW) convolution layers to replace the linear projection layers in the original FFN. This change enhances the model capacity to capture and process patterns, particularly in understanding the spatial relationships among local features. Following the output from the SCT layer, the input to the DW-FFN (Fig. 4(A2)) has the shape of HW × C. Before processing, the input is reshaped from HW × C to H × W × C. The DW-FFN can be written as:

(4) $$\text{Y}=\text{Con}{\text{v}}_{\text{P}{\text{W}}_{2}}(\text{GeLU}(\text{DW}(\text{Con}{\text{v}}_{\text{P}{\text{W}}_{1}}({\text{x}}_{\text{input}}))))$$

where $\text{Con}{\text{v}}_{\text{P}{\text{W}}_{1}}$ and $\text{Con}{\text{v}}_{\text{P}{\text{W}}_{2}}$ are the convolution layers with a kernel size of 1 × 1, a stride of 1, the filter size of $\text{Con}{\text{v}}_{\text{P}{\text{W}}_{1}}$ is 4C, and the filter size of $\text{Con}{\text{v}}_{\text{P}{\text{W}}_{2}}$ is C. DW is a depth-wise convolution layer with a kernel size of 3 × 3, and a stride of 1.

#### 4.2.3. Squeeze-and-excitation (SE) block

As a plug-and-play module, the SE block (Fig. 4(B)) [50] enhances feature extraction by mapping channel relationships in convolutional features, thereby improving the representation of complex patterns. In addition, the SE block enhances network performance by adaptively recalibrating functions with minimal computational cost and without major architectural changes. Hence, the SE block is utilized to enhance the features from the skip connection for feature fusion. Assume the input to the SE block has a shape of H × W × C. After processing by the global average pooling (GAP) layer, the output shape becomes 1 × 1 × C. In this study, the first linear projection layer reduces this to C/8 units, and the second linear projection layer restores it to C units. Finally, the sigmoid activation layer outputs a shape of 1 × 1 × C. By performing element-wise multiplication with the original input of the SE block, the forward processing is completed.

#### 4.2.4. Linear decoder block

Xie et al. [38] result has shown that the linear projection (LP) layer only segmentation decoder can reduce model complexity while providing a larger effective receptive field than CNN decoder. Thus, we propose a linear decoder (Fig. 4(C)) that only contained the LP layer to cascade upsample the feature map for the segmentation decoder. The linear decoder consists of two LP layers, a GeLU activation layer and a layer normalization (LN) layer, and the forward processing can be written as:

(5) $$\text{Y}=\text{L}{\text{P}}_{2}(\text{GeLU}(\text{LN}(\text{L}{\text{P}}_{1}(\text{x}))))$$

where x is the input of the linear decoder block and with a shape of H × W × C, the $\text{L}{\text{P}}_{1}$ has a unit of C/2 and $\text{L}{\text{P}}_{2}$ has a unit of C.

### 4.3. Loss function

In this study, two loss functions are utilized to calculate the loss for MTDL model optimization, including denoising loss $({\text{L}}_{\text{D}})$ and segmentation loss $\left({\text{L}}_{\text{seg}}\right)$ . Based on our previous works [52], the combination of mean squared error (MSE) and VGG19-based content loss can improve the OCT image denoising performance. Assume the I is the ground-truth image and $\hat{I}$ is the denoised image from ESDM, the equation of the denoising loss can be written as:

(6) $${\text{L}}_{\text{D}}(\text{I},\hat{I})=\alpha *\sum_{\text{i}=1}^{\text{N}}{\left({\text{I}}_{\text{i}}-{\hat{I}}_{\text{i}}\right)}^{2}+\beta *\sum_{\text{i}=1}^{\text{N}}{\left(\emptyset ({\text{I}}_{\text{i}})-\emptyset ({\hat{I}}_{\text{i}})\right)}^{2}$$

where N is the pixel number in the image, ∅ is the ImageNet2 K data pre-trained VGG19 model for feature map extraction, α and β are hyper-parameters to control the weights of ${\text{L}}_{\text{D}}$ .

Regarding the loss function to calculate the error between the predicted mask and the pseudo mask generated by the pre-trained model, the cross-entropy loss function is used. In this study, the cross-entropy loss function, denoted as ${\text{L}}_{\text{seg}}$ , is selected for its effectiveness in pixel-wise classification tasks, enabling precise evaluation of the prediction accuracy by comparing the predicted probabilities with the actual class labels at each pixel. Considering the precious of the pseudo label is not as high as the manual label, the soft-label approach with a confidence level of 0.95 was utilized in the cross-entropy loss function. Taking each pixel *i* of the mask with true class *c*, in a 3-class segmentation task, the soft labels can be defined as: $y_{i,c}=0.95$ for the true class, and $y_{i,k}=\frac{0.05}{2}=0.025$ for the other two classes k≠c , the equation is shown as:

(7) $${\text{L}}_{\text{Seg}}=-\frac{1}{\text{N}}\sum_{i=1}^{N}\left(0.95\;\log({\hat{y}}_{i},true)+0.025\sum_{C\neq true}\text{log}({\hat{y}}_{i},c)\right)$$

where N is the total number of the pixels in the mask, ${\hat{y}}_{i,c}$ is the predicted probability of pixel *i* belong to class *c*. By using the soft-label approach, the loss function becomes more tolerant to slight inaccuracies, and improves the overall stability and performance of the model. The combined loss function for MTDL training is then shown as:

(8) $${\text{L}}_{\text{c}}={\text{L}}_{\text{D}}+{\text{L}}_{\text{seg}}$$

## 5. Experiment and results

### 5.1. Implementation details

The ESDM proposed in this study was built and trained based on TensorFlow 2.9.0. Based on our previous experiment [52,53], the α and β were set as 1 and 0.01 in Eq. (6) to provide the best image denoising performance. The Adam optimizer with a learning rate of 0.001 was used to update the trainable parameters in the model [54]. The number of training epoch was set as 200 with a batch size of 16. An NVIDIA RTX 4090 with 24 GB memory was utilized to facilitate the model training. A checkpoint saving strategy was used to save the model weights that have the lowest validation loss.

Regarding the initialization of the ESDM, as shown in Fig. 3, the filter size ( $FS_{i}$ , where i∈{1,2,3,4} ) of the SCT blocks, linear decoders, and convolution blocks at each stage are {32, 64, 128, 256, 256}, the number of SCT block ( $N_{i}$ , where i∈{1,2,3} ) at each stage are {1, 2, 2}, and the number of heads ( $nH_{i}$ , where i∈{1,2,3} ) at each SCT blocks are {4, 8, 8}. Expect the convolution layer which highlights with a kernel size of 1 × 1, all convolution layers, depth-wise convolution layer, and deconvolution layer have a kernel size of 3 × 3. In terms of the upsampling operations, deconvolution layers with a stride of 2 are used in the image denoising decoder, and an efficient bilinear interpolation method is used in the image segmentation decoder. For the output layers, both image segmentation and denoising decoders utilize a convolution layer with a kernel size of 3 × 3 and a stride of 1, while the filter size is 1 for denoising and 3 for segmentation, respectively.

### 5.2. Evaluation metrics

To quantify the performance among various methods mentioned in this study, we utilized peak signal-to-noise ratio (PSNR) and structural similarity (SSIM) [55] for image denoising metrics. Then, two additional metrics for image segmentation, including mean intersection over union (mIoU) and mean dice similarity coefficients (mDice). Those metrics can be formulated as:

(9) $$\text{PSNR}(\text{y},\hat{y})=10\log_{10}\left(\frac{{\text{I}}_{\text{max}}^{2}}{\text{MSE}(\text{y},\hat{y})}\right)$$

where ${\text{I}}_{\text{max}}$ is the maximum value of the pixel in the image, MSE is the mean squared error result between the high-quality reference image y and denoised image $\hat{y}$ .

(10) $$\text{SSIM}(\text{y},\hat{y})=\frac{\left(2\mu_{\text{y}}\mu_{\hat{y}}+{\text{C}}_{1})(2\sigma_{y\hat{y}}+{\text{C}}_{2}\right)}{\left(\mu_{\text{y}}^{2}+\mu_{\hat{y}}^{2}+{\text{C}}_{1})(\sigma_{\text{y}}^{2}+\sigma_{\hat{y}}^{2}+{\text{C}}_{2}\right)}$$

where $\mu_{\text{y}}$ and $\mu_{\hat{y}}$ are the mean intensities of the high-quality reference image y and denoised image $\hat{y},\sigma_{\hat{y}}^{2}$ and $\sigma_{\hat{y}}^{2}$ are the variances of y and $\hat{y},\sigma_{y\hat{y}}$ is the covariance of the y and $\hat{y}$ , and ${\text{C}}_{1}$ and ${\text{C}}_{2}$ are constants to stabilize the division.

(11) $$\text{mIoU}=\frac{1}{\text{K}}\sum_{\text{i}=1}^{\text{K}}\frac{\text{T}{\text{P}}_{\text{i}}}{\text{T}{\text{P}}_{\text{i}}+\text{F}{\text{P}}_{\text{i}}+\text{F}{\text{N}}_{\text{i}}}$$

(12) $$\text{mDice}=\frac{1}{\text{K}}\sum_{\text{i}=1}^{\text{K}}\frac{2\times \text{T}{\text{P}}_{\text{i}}}{2\times \text{T}{\text{P}}_{\text{i}}+\text{F}{\text{P}}_{\text{i}}+\text{F}{\text{N}}_{\text{i}}}$$

where TP is the true position and represents the number of pixels correctly predicted and labeled as positive. Conversely, the number of pixels that are incorrectly given a positive label is called FP. FN represents the false negative, and K is the number of classes.

### 5.3. Measurement of epithelium thickness

To measure the thickness of the epithelium, the epithelium segmentation distance in pixels and the conversion coefficient of the OCT imaging were multiplied. As mentioned in section 3.1, the conversion coefficient in air is ∼8.74 µm/pixel. Based on the Jacques [56] experiments, the refractive index of the oral tissue typically ranges from 1.38 to 1.41. Assuming an average refractive index of 1.395 for human oral tissue, the conversion coefficient in human oral tissue can be calculated as ∼6.27 µm/pixel (8.74/1.395). Hence, the real thickness of the human oral epithelium can be measured and calculated using the following formulation.

(13) $$\text{Epithelium Thickness}={\text{N}}_{\text{pixel}}\times \frac{\text{Axial Resolution}}{\text{n}}$$

where ${\text{N}}_{\text{pixel}}$ is the number of pixels between the segmented boundaries of the epithelium layer, n is refractive index of oral tissue (1.395), and axial resolution in this system is ∼8.74 µm/pixel.

### 5.4. Comparison to other neural networks

A series of models are utilized to compare with the ESDM, including SRResNet [23], SwinIR [34], U-Net [24], SegFormer [38], SHFormer [45], Uformer [35], LU-Swin-Transformer (LU-Swin-T) [52], TransUNet [36], and SwinUNet [37]. Among them, SRResNet, SwinIR and UFormer are specifically designed for image denoising and are only used in image denoising comparison. SegFormer and SHFormer, which have asymmetric architectures, are only used in image segmentation comparison. U-Net, TransUNet, Swin-UNet, and LU-Swin-T are used for both segmentation and denoising performance comparison. In addition, to evaluate the impact of the multi-task architecture, we also built multi-task versions of TransUNet, U-Net, Swin-UNet, and LU-Swin-T by copying the same decoder for segmentation and denoising.

The training strategies and details of the compared models are the same as the proposed implementation details mentioned above to minimize training influence. We also provide a comparison related to parameters and floating points operation (FLOPs), representing the model memory occupation and computational demand, respectively. The model parameters and FLOPs are calculated based on an input shape with 256 × 256 × 1.

#### 5.4.1. Quantitative comparison with other methods

Table 1 is a quantitative comparison between different methods in oral OCT image denoising and segmentation. In denoising task, compared to the input image generated from one repetition of OCT scan, all deep-learning models enhance both PSNR and SSIM performance. Among them, our ESDM offers the best denoising performance in terms of PSNR (26.272) and SSIM (0.737), with model complexity (FLOPs: 9.84 G) being the second smallest among all image denoising models and the smallest among all multi-task models.

In terms of the segmentation task, both ESDM and SwinUNet (single task) have the highest mDice score (0.972). Besides, the ESDM has the highest mIoU score of 0.948. While SwinUNet (single task) presents a similar performance to ESDM, the ESDM has relatively lower model complexity (FLOPs: 9.84 G < 29.65 G). The SHFormer has the smallest model complexity (1.09 G) while providing the second-highest mDice (0.970) and third-highest mIoU (0.946). However, SHFormer is specifically designed for image segmentation task and cannot simultaneously achieve image denoising.

**Table 1.** Quantitative Comparison (Mean ± Standard Deviation) with Other Models in Image Segmentation and Denoising. Param: Network Parameters

**Methods**	**Task Definition**	**Image Denoising**		**Image Segmentation**		**Param ↓**	**FLOPs ↓**
		**PSNR ↑**	**SSIM ↑**	**mDice ↑**	**mIoU ↑**		
**Noisy Input**	N/A	20.704 ± 1.035	0.675 ± 0.075	N/A	N/A	N/A	N/A
**SRResNet**	Denoising	25.386 ± 0.710	0.715 ± 0.035	N/A	N/A	0.716 M	93.49 G
**SwinIR**	Denoising	25.771 ± 1.274	0.735 ± 0.054	N/A	N/A	2.084 M	183.93 G
**UFormer**	Denoising	26.269 ± 1.031	0.736 ± 0.037	N/A	N/A	24.651 M	72.90 G
**SegFormer**	Segmentation	N/A	N/A	0.969 ± 0.034	0.944 ± 0.056	3.702 M	3.49 G
**SHFormer**	Segmentation	N/A	N/A	0.970 ± 0.042	0.946 ± 0.065	2.817 M	1.09 G
**LU-Swin-T**	Denoising	25.797 ± 0.899	0.704 ± 0.049	N/A	N/A	11.976 M	7.21 G
	Segmentation	N/A	N/A	0.967 ± 0.042	0.940 ± 0.065	11.976 M	7.22 G
	Multi-task	25.183 ± 1.083	0.722 ± 0.042	0.967 ± 0.038	0.940 ± 0.059	14.839 M	10.89 G
**U-Net**	Denoising	25.017 ± 0.935	0.672 ± 0.024	N/A	N/A	34.566 M	106.45 G
	Segmentation	N/A	N/A	0.968 ± 0.026	0.940 ± 0.045	34.567 M	106.67 G
	Multi-task	25.046 ± 0.755	0.685 ± 0.031	0.969 ± 0.030	0.942 ± 0.050	50.242 M	174.36 G
**TransUNet**	Denoising	24.101 ± 0.899	0.677 ± 0.050	N/A	N/A	52.351 M	41.38 G
	Segmentation	N/A	N/A	0.945 ± 0.074	0.906 ± 0.107	52.353 M	41.54 G
	Multi-task	24.742 ± 0.924	0.684 ± 0.039	0.944 ± 0.064	0.903 ± 0.093	64.924 M	65.71 G
**SwinUNet**	Denoising	26.160 ± 0.996	0.718 ± 0.046	N/A	N/A	50.399 M	29.32 G
	Segmentation	N/A	N/A	0.972 ± 0.027	0.947 ± 0.045	50.402 M	29.65 G
	Multi-task	25.681 ± 0.885	0.709 ± 0.036	0.968 ± 0.039	0.941 ± 0.061	61.487 M	43.08 G
**ESDM**	Multi-task	26.272 ± 0.965	0.737 ± 0.042	0.972 ± 0.035	0.948 ± 0.055	7.693 M	9.84 G

#### 5.4.2. Visual comparison with other methods

**Fig. 5.**
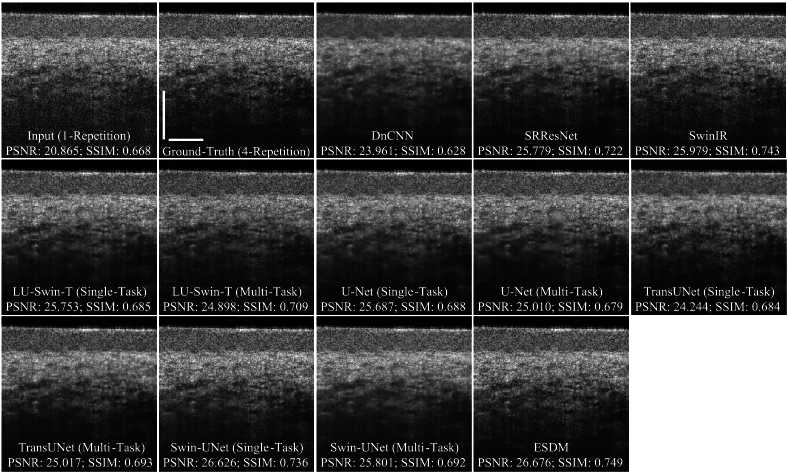
The visual comparison of image denoising. The white scale bar is 500 µm. The scan area is the oral lip with the LSM03 sample lens.

Figure 5 is the visual comparison of oral lip OCT image denoising performance among deep-learning models. All models enhance PSNR and SSIM performance. Compared to the high-quality ground-truth images, the ESDM provides the highest PSNR (26.676) and SSIM (0.749). In the visual comparison, all neural network methods can reduce the noise and enhance the image contrast in the denoised OCT images.

**Fig. 6.**
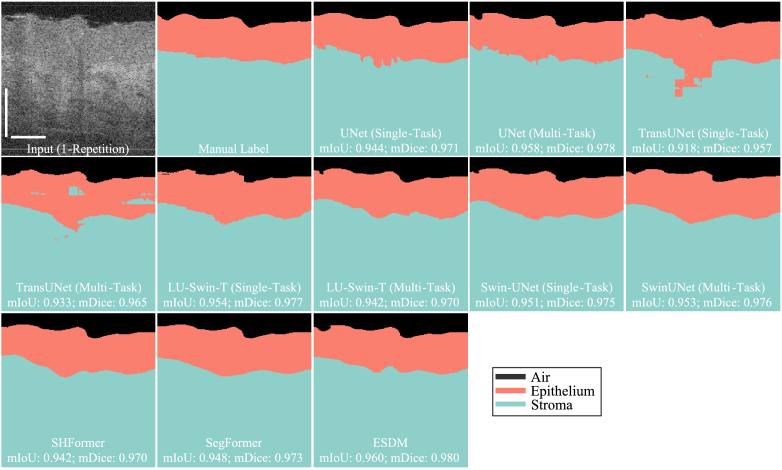
The visual comparison of image segmentation. The white scale bar is 500 µm. The scan area is the intraoral side with the oral probe sample lens.

Figure 6 is a visual comparison of the image segmentation results from various models. The mIoU and mDice are calculated based on the comparison with the manual label. Among all the models evaluated, our proposed method, ESDM, achieves the highest mIoU (0.960) and mDice (0.980) scores, demonstrating a superior overlap and similarity with manual labels.

### 5.5. Ablation studies

#### 5.5.1. Influence on feature fusion strategies

Table 2 presents the performance of the ESDM with various feature fusion strategies, including the proposed architecture (using the skip connection with SE-block), without the skip connection, and with the skip connection but without the SE-block. The results show that the proposed ESDM can provide the best image denoising and segmentation performance.

**Table 2.** Quantitative Comparison (mean ± standard deviation) on Feature Fusion Strategies

Fusion Strategies		Image Denoising		Image Segmentation		Parameter ↓	FLOPs ↓
SE Block	Skip	PSNR ↑	SSIM ↑	mDice ↑	mIoU ↑		
√	√	26.272 ± 0.965	0.737 ± 0.042	0.972 ± 0.035	0.948 ± 0.055	7.693 M	9.84 G
×	√	26.202 ± 0.956	0.736 ± 0.041	0.970 ± 0.040	0.945 ± 0.062	7.671 M	9.83 G
×	×	23.382 ± 1.208	0.691 ± 0.075	0.973 ± 0.029	0.949 ± 0.047	7.543 M	9.16 G

Notably, the ESDM without using the skip connection significantly decreases image denoising performance (PSNR: 23.382 < 26.272; SSIM: 0.691 < 0.737). The model complexity comparison shows that the skip connection and SE-block only slightly increase model parameters (from 7.543 M to 7.693 M) and complexity (from 9.16 G to 9.84 G).

#### 5.5.2. Influence on loss function

Table 3 presents the comparison of the ESDM under training with various loss functions, indicating that the proposed ${\text{L}}_{\text{D}}$ in Eq. (6) enhances both segmentation and denoising performance (PSNR: 26.082 < 26.272; SSIM: 0.720 < 0.737; mDice: 0.970 < 0.972; mIoU: 0.946 < 0.948).

**Table 3.** Quantitative Comparison (mean ± standard deviation) on Loss Function

Loss Function		Image Denoising		Image Segmentation		Parameter ↓	FLOPs ↓
MSE	Content Loss	PSNR ↑	SSIM ↑	mDice ↑	mIoU ↑		
√	√	26.272 ± 0.965	0.737 ± 0.042	0.972 ± 0.035	0.948 ± 0.055	7.693 M	9.84 G
√	×	26.082 ± 0.896	0.720 ± 0.043	0.970 ± 0.043	0.946 ± 0.066	7.693 M	9.84 G

#### 5.5.3. Component-wise ablation

In this section, we evaluate the efficiency of the multi-task design of ESDM by disabling specific decoders and leading the ESDM as a single-task model. Table 4 shows the comparison between various settings, including multi-task, denoising only, and segmentation only. In terms of image denoising, the multi-task design achieves higher PSNR (26.272 > 26.1) and SSIM (0.737 > 0.721) performance while only increasing parameters by 0.104 M and FLOPs by 0.1 G. In the image segmentation task, the multi-task design provides higher mDice (0.972 > 0.970) and mIoU (0.948 > 0.945). Based on this ablation study, we believe that the ESDM with a multi-task design can enhance performance by sharing features from the shared encoder while increasing model efficiency.

**Table 4.** Quantitative Comparison (mean ± standard deviation) on Model Component-wise

Task Definition	Image Denoising		Image Segmentation		Parameter ↓	FLOPs ↓
	PSNR ↑	SSIM ↑	mDice ↑	mIoU ↑		
Multi-task	26.272 ± 0.965	0.737 ± 0.042	0.972 ± 0.035	0.948 ± 0.055	7.693 M	9.84 G
Reconstruction	26.100 ± 1.195	0.721 ± 0.044	N/A	N/A	7.589 M	9.74 G
Segmentation	N/A	N/A	0.970 ± 0.033	0.945 ± 0.053	4.578 M	3.89 G

#### 5.5.4. Influence on filter size

As mentioned in section 5.1, the original settings of filter sizes in ESDM are {32, 64, 128, 256, 256}. In this section, we change the network size by increasing and decreasing the filter sizes to evaluate the influence on model size. Table 5 shows the results from various filter sizes of ESDM. In image segmentation, decreasing the filter size decreases performance, in terms of mDice (0.971 < 0.972) and mIoU (0.945 < 0.948), while increasing the filter size does not improve image segmentation performance. In image denoising, increasing the filter size increases PSNR performance, from 26.179 to 26.325. However, increasing the filter size from {32, 64, 128, 256, 256} to {48, 96, 192, 384, 384} does not improve SSIM. Considering the balance of model inference, image denoising, and segmentation performance, we use the proposed filter size in this study.

**Table 5.** Quantitative Comparison (mean ± standard deviation) on Filter Size

Filter Sizes	Image Denoising		Image Segmentation		Parameter ↓	FLOPs ↓
	PSNR ↑	SSIM ↑	mDice ↑	mIoU ↑		
{16, 32, 64, 128, 128}	26.179 ± 1.075	0.725 ± 0.051	0.971 ± 0.044	0.945 ± 0.064	1.959 M	2.89 G
{32, 64, 128, 256, 256}	26.272 ± 0.965	0.737 ± 0.042	0.972 ± 0.035	0.948 ± 0.055	7.693 M	9.84 G
{48, 96, 192, 384, 384}	26.325 ± 0.996	0.737 ± 0.041	0.972 ± 0.028	0.947 ± 0.047	17.203 M	20.90 G

### 5.6. Model inference complexity comparison

**Fig. 7.**
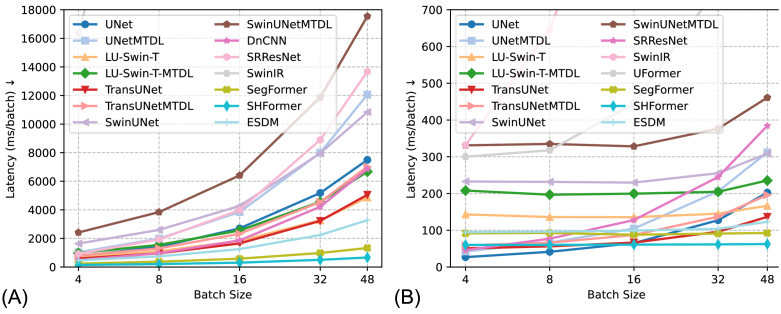
Comparison of Model Inference under Various Batch Size on (A) CPU and (B) GPU. The results from SwinIR are so large that it would negatively influence the visualization in the chart, therefore we decided to limit the y-axis of (A) from 0 to 18000 and (B) from 0 to 700. The inference times (unit: ms) of SwinIR (under batch sizes 4, 8, 16, 32, 48) are {16362, 36923, 73339, 141759, 212695} in (A) CPU and {331.8, 646.1, 1261.8, 2500.3, 3744.3} in (B) GPU.

Figure 7 is the comparison of the model inference under various batch size settings and different hardware environments, including CPU and GPU. The testing platform includes a CPU (Intel i9-12900 K), GPU (Nvidia RTX 4090), and 64 GB of memory. In both CPU-only and GPU-accelerated environments, the SHFormer with the smallest FLOPs (1.09 G) has the lowest inference time, and our proposed ESDM has the smallest inference time among models designed for multi-tasks. Among models used in image denoising tasks, our proposed ESDM achieves the lowest inference time under both CPU and GPU.

### 5.7. Oral epithelium thickness quantification

Based on the segmentation mask manually labeled by experts, we evaluate the precision of the segmentation results from the ESDM. The thickness results are shown in Table 6. The method for epithelium thickness calculation is mentioned in section 5.3. The data utilized in this section is mentioned in section 3.3. In addition, the mean-absolute-error (MAE) is utilized to quantify the difference between the manually measured thickness and that obtained through deep learning-based methods.

**Table 6.** The Oral epithelium thickness quantification (mean ± standard deviation) based on manual and deep-learning-based methods. Seg represents segmentation-only architecture, and MT is multi-task architecture

Methods		Oral Lip		Oral Side	
		Intraoral Lens	LSM03	Intraoral Lens	LSM03
**Manual**	Thickness	280.53 ± 91.60	253.95 ± 43.93	322.86 ± 32.20	348.69 ± 29.92
**ESDM**	Thickness	272.83 ± 90.60	256.28 ± 41.14	320.17 ± 33.10	352.49 ± 32.98
	MAE	9.00 ± 7.53	6.18 ± 5.35	5.40 ± 3.69	10.33 ± 9.28
**SegFormer**	Thickness	275.15 ± 89.03	255.59 ± 42.12	322.91 ± 34.05	346.20 ± 31.00
	MAE	8.24 ± 5.67	6.64 ± 5.36	6.90 ± 5.93	8.22 ± 8.56
**SHFormer**	Thickness	272.46 ± 88.89	256.85 ± 41.57	320.50 ± 33.47	349.92 ± 33.80
	MAE	9.66 ± 8.04	7.00 ± 6.98	6.23 ± 4.21	11.95 ± 8.47
**LU-Swin-T (Seg)**	Thickness	284.10 ± 97.45	233.40 ± 49.31	307.92 ± 39.37	332.34 ± 35.74
	MAE	26.28 ± 25.70	23.10 ± 46.52	19.27 ± 17.82	26.02 ± 22.46
**LU-Swin-T (MT)**	Thickness	267.62 ± 89.97	248.36 ± 41.55	313.69 ± 34.78	339.94 ± 31.70
	MAE	13.56 ± 11.24	10.86 ± 17.57	10.04 ± 6.88	13.94 ± 13.58
**SwinUNet (Seg)**	Thickness	271.62 ± 92.46	244.51 ± 46.30	318.22 ± 35.42	303.77 ± 45.16
	MAE	11.56 ± 8.28	15.50 ± 24.66	8.49 ± 7.82	48.07 ± 36.32
**SwinUNet (MT)**	Thickness	269.33 ± 89.15	241.06 ± 46.45	318.76 ± 34.84	294.68 ± 61.10
	MAE	15.87 ± 14.09	19.35 ± 26.31	10.61 ± 10.06	56.90 ± 55.03
**TransUNet (Seg)**	Thickness	195.06 ± 41.31	224.24 ± 37.01	309.07 ± 32.68	294.63 ± 36.40
	MAE	86.26 ± 97.78	33.08 ± 46.14	19.53 ± 15.95	54.06 ± 31.58
**TransUNet (MT)**	Thickness	194.79 ± 46.18	237.43 ± 41.60	247.24 ± 28.54	297.62 ± 42.66
	MAE	85.74 ± 77.75	18.79 ± 19.22	75.61 ± 31.41	51.07 ± 28.52
**U-Net (Seg)**	Thickness	328.92 ± 127.82	259.04 ± 43.68	352.88 ± 38.55	352.62 ± 31.86
	MAE	17.21 ± 9.23	6.74 ± 5.42	12.16 ± 6.71	9.48 ± 8.94
**U-Net (MT)**	Thickness	264.98 ± 92.10	246.53 ± 41.53	309.78 ± 36.94	338.31 ± 37.08
	MAE	15.92 ± 10.21	11.43 ± 15.17	15.20 ± 9.87	16.42 ± 15.12

For the oral lip region, ESDM has the lowest mean MAE (6.18) with a measured thickness of 256.28 ± 41.14 in the data from LSM03. Besides, SegFormer has the lowest mean MAE of 8.24, and ESDM is the second-lowest (mean MAE: 9.00) in the data from the intraoral lens. For the oral side region, ESDM has achieved the lowest mean MAE of 5.4 in the data from the intraoral lens (thickness: 320.17 ± 33.10), and the third-lowest mean MAE of 10.33 in the data from LSM03. In addition, SHFormer has the second-lowest mean MAE of 6.23 in the data from the intraoral lens, and SegFormer has the lowest mean MAE of 8.22 in the LSM03 data.

The accuracy of measured thickness among various deep-learning-based methods is highly similar to the mDice and mIoU performance presented in Table 1. In particular, ESDM and SegFormer demonstrate consistent performance across both the intraoral lens and LSM03 data, indicating robust generalization capabilities. SHFormer also has promising results, especially in the oral side region of intraoral lens data.

## 6. Discussion

In this study, we present the ESDM to facilitate the OCT scan (from ∼8s to ∼2s), reduce unpredictable motion artifacts during the OCT scan, and enhance the efficiency of oral mucosal layer segmentation while maintaining OCT imaging quality of oral tissues. Combining local feature extraction from convolution layers with the long-term information processing of transformer layers, ESDM improves image segmentation and denoising performance compared to existing models. With the ablation studies and model inference comparison, we further prove that the proposed ESDM is efficient for oral OCT image processing. Besides, the results in Table 6 indicate that ESDM has high accuracy in epithelium thickness quantification.

To verify the performance of the ESDM, we conducted a full comparison with a series of existing state-of-the-art models. The results in Table 1, indicating that ESDM has the best image segmentation and denoising performance (mDice: 0.972, mIoU: 0.948, PSNR: 26.272, SSIM: 0.737). Based on the visual comparison, the proposed ESDM has the best performance to adapt to segmentation and denoising for oral tissue. Moreover, the results from the ablation studies (from Table 2 to Table 5) indicate that the proposed ESDM architecture and loss function can mostly increase the segmentation and denoising performance while maintaining the model efficiency, e.g., model FLOPs. Additionally, the epithelium thickness measurement results in Table 6 that ESDM consistently achieves lower MAE and higher accuracy in thickness measurements, making it highly preferable for clinical applications where precision is critical.

This study has significant clinical relevance by expanding the application of OCT in addition to ophthalmology to the oral cavity. Currently, OCT is rarely used to examine soft tissues such as the intraoral region and lips. By demonstrating precise measurement of oral epithelium thickness, this research facilitates early detection and diagnosis of oral pathologies like dysplasia and oral cancer. Integrating standardized oral OCT scanners into clinical workflows can enhance non-invasive diagnostic capabilities, enabling clinicians to perform real-time assessments and make informed treatment decisions. Future research could explore flexible OCT scans covering a wider range and apply the method reported here to standardize oral OCT scanners for precise oral epithelium thickness measurement.

Our study has limitations. Firstly, the data used for training the ESDM model were from healthy participants, which may affect the performance when applied to data from subjects with oral diseases. Future work will involve collecting OCT data from individuals with various oral conditions to enhance the robustness of the multi-task pipeline. Secondly, the performance of the model might be degraded when facing the data with serious motion artifacts. Future work will try to train the model with serious motion artifact data. Thirdly, although we examined our methods using data collected from two sample lenses, reliance on a single OCT device for data collection may limit the applicability of ESDM in environments with different OCT devices, especially commercial devices. Future studies should include more data from a broader range of OCT devices, pathologies, and age groups to ensure wider applicability.

## 7. Conclusion

In conclusion, the proposed ESDM provides a significant advance in OCT imaging of oral mucosal tissues, offering a computationally efficient tool for non-invasive analysis that enables high-precision segmentation and improves image quality with a fast single OCT scan. ESDM performs well in denoising and segmentation tasks, making it valuable for clinical applications. Future studies could further optimize ESDM and explore its application in other imaging modalities, potentially expanding its impact in medical imaging.

## Data Availability

Data and Python code underlying the results presented in this paper are not publicly available at this time but may be obtained from the authors upon reasonable request.
